# Is it inevitable that societies exploit useful technologies?

**DOI:** 10.3389/fpsyg.2014.00224

**Published:** 2014-03-13

**Authors:** David Trafimow

**Affiliations:** Department of Psychology, New Mexico State UniversityLas Cruces, NM, USA

**Keywords:** technology, comparative history, stirrup, gunpowder, China

In some applied areas of psychology—particularly engineering psychology, human factors psychology, and related areas—a common underlying assumption is that it is inevitable that people will seize upon useful new technologies and exploit them with dispatch, though some have suggested more complex points of view (Davis, 1993). In turn, useful technologies transform human societies (Woolgar, 2009). It is easy to provide examples that confirm this assumption. Furthermore, it is difficult to list counterexamples, which serves to further fix the assumption in the minds of those researchers who are at the intersection of humans and technology.

There is a good reason why it is difficult to list counterexamples. Consider that for us to agree that a technology is useful at the larger level of the whole society (rather than just specialized uses that have little impact on the larger society), that technology needs to be demonstrated to have been used at a societal level or else there is no way for us to know that it is, in fact, useful at that level. Obviously, if the technology has been used successfully—again, this is a prerequisite for us to know that it is useful—it would seem to be a tautology that the technology has been used successfully! Is there a way out of the tautology? Is there a way to test, independently, whether a technology is useful and whether it has been used?

A consideration of history suggests a possible way out. An advantage of studying the history of different cultures is that they can serve as naturally occurring “control” conditions for each other. For example, historians credit two technological innovations with being responsible for much of the history of Western Civilization. One of these is the popularization of the stirrup in the medieval centuries in Europe, which significantly altered mounted warfare by greatly reducing the likelihood of a rider falling off of his horse while fighting, increasing the force and variety with which mounted riders could strike blows, increasing the efficacy of shock tactics, and others. Some authorities have even gone so far as to credit the stirrup with being largely responsible for the advent of feudalism (e.g., White, 1962):

“Few inventions have been so simple as the stirrup, but few have had so catalytic an influence on history. The requirements of the new mode of warfare which it made possible found expression in a new form of western European society dominated by an aristocracy of warriors endowed with land so that they might fight in a new and highly specialized way” (p. 38).

Another example is the popularization of guns and gunpowder during the late middle age and renaissance in Europe. The military uses and effects on history are too well known to necessitate further elaboration here. There can be little doubt that the stirrup and gunpowder both had an enormous influence on the history of Western Civilization.

Thus far, the stirrup and gunpowder seem to be excellent examples of humans exploiting advances in technology. But the picture changes substantially if we switch our focus to the history of China, whose societies failed to exploit these technologies at a level commensurate with European exploitation. The historian, Dreyer (2002), both acknowledged the importance of the stirrup and gunpowder in Europe while denying equal importance in China:

“China's long history of technological progress provides scant comfort for theories that see certain kinds of social and political change as the inevitable result of specific technologies. Neither the stirrup nor gunpowder had the dramatic consequences in China claimed for them in Europe” (pp. 28–29).

It is important to be clear that Dreyer was not claiming that the Chinese did not have the stirrup or gunpowder because they did, and earlier than the Europeans had them. Nor was Dreyer claiming that the Chinese made no use whatsoever of these technologies. Rather, Dreyer's point is that the technologies did not have the transformative effects that historians have pointed to in the history of Western Civilization. There are many arguments that can be made to explain this difference between West and East, but for my purpose, the mere historical fact of the difference between West and East is sufficient.

So how do the examples of the stirrup and gunpowder enable researchers to circumvent the foregoing tautology? The answer is that both technologies were proven to be extremely useful and exploitable in the West. Nevertheless, their effects on society in the East were greatly attenuated. What happened in the West gives independent evidence of the potential utility of the technologies whereas their relative lack of use in the East demonstrates that it is not the case that people will necessarily rush to use technologies that are new and useful. Put another way, the stirrup and gunpowder provide two devastating examples from history that contradict the typical assumption made by applied researchers at the intersection of psychology and technology that the advent of new and useful technologies inevitably leads to their widespread exploitation and associated societal transformations (Davis, 1993).

A possible counter to my argument might be that the stirrup and gunpowder were useful in the West and not in the East, and for that reason were adopted in the former but not in the latter area. However, the historical record indicates otherwise. The Mongols went through China like a hot knife through butter under their great leader Genghis Khan, and they were aided greatly by the fact that they had stirrups (De Hartog, 1989). In large part it was the stirrup that enabled them to conquer China and eventually establish the Yuan dynasty. This is strong evidence that stirrups were important in the East, or at least would have been if people had recognized the possibilities, but the historical fact of the matter is that they were way behind the West in exploiting the technology.

The same is true of gunpowder. Although the East was far behind the West in developing the potentialities of the technology, once they saw it, they did adopt it (Lococo, 2002). During the transition period, some of the Chinese hired Western cannon experts, and thereby had an important advantage against Chinese who did not do that. So the potential importance of gunpowder eventually became obvious even in the East, particularly during the Qing dynasty (Lococo, 2002).

In summary, stirrups and gunpowder would have been extremely important in the East, as well as in the West, if only the people in the East had seen (and developed) the possible ways to exploit the technologies as people in the West did. But the historical fact is that they did not. This is not because these ways of exploiting the technologies were unimportant in the East, relative to the West, but rather because people just did not see them. Possibly, this is because of cultural prejudices. In China, many saw no reason to change the eternal “Kingdom of Heaven” and in Japan, the Samurai resisted the switch to gunnery (Perrin, 1979; Dreyer, 2002). Thus, we see that societies do not always adopt useful technologies. This is not necessarily due to the technologies being potentially more important in one culture than in another, but rather that cultures themselves can blind people to possibilities as well as make people aware of possibilities.

In conclusion, the examples of the stirrup and gunpowder show that it is a fallacy to automatically assume that useful new technologies will be exploited in a timely fashion. They also illustrate the value of psychologists looking beyond the psychology literature, which is biased in a Western direction, to consider what has been discovered by researchers in other social sciences, such as history.
